# A Complete Feasible and Nodes-Grouped Scheduling Algorithm for Wireless Rechargeable Sensor Networks in Tunnels

**DOI:** 10.3390/s18103410

**Published:** 2018-10-11

**Authors:** Xiaoming Liu, Yu Guo, Wen Li, Min Hua, Enjie Ding

**Affiliations:** 1 College of Information Science and Technology, Nanjing Forestry University, Nanjing 210037, China; lxm@njfu.edu.cn (X.L.); min_hua@njfu.edu.cn (M.H.); 2 IOT Perception Mine Research Center, School of Information and Control Engineering, China University of Mining and Technology, Xuzhou 221116, China; guo_yu@cumt.edu.cn; 3 School of Information Engineering, Nanjing Audit University, Nanjing 211815, China; liwen0779@nau.edu.cn

**Keywords:** WSNs, WRSNs, charging scheduling, tunnels

## Abstract

Limited energy in each node is the major design constraint in wireless sensor networks (WSNs), especially in mine tunnel scenario where the WSNs are required to work perpetually. To overcome this limit, wireless rechargeable sensor networks (WRSNs) have been proposed and studied extensively over the last few years. To keep the sensor nodes working perpetually, one fundamental question is how to design the charging scheme. Considering the special tunnel scenario, this paper proposes a Complete Feasible Charging Strategy (CFCS) to ensure the whole WRSNs is working perpetually. We divide the whole WRSN into several subnetworks and use several mobile chargers (MCs) to charge every subnetwork periodically and orderly. For a subnetwork, we formulate the main problem as a charging time distribution problem. A series of theorems are deduced to restrict the charging configurations, and a group nodes mechanism is proposed to expand the scale of the WRSNs. Finally, we conduct extensive simulations to evaluate the performance of the proposed algorithms. The results demonstrate which of the CFCS boundary theorems is correct and that our proposed CFCS can keep the WRSNs working perpetually. Furthermore, our Nodes-Grouped mechanism can support more nodes in WRSN compared to the state-of-the-art baseline methods.

## 1. Introduction

Nowadays, wireless sensor networks (WSNs) are playing a significant role in mine tunnels for monitoring gas, rock pressure, temperature, personnel orientation, etc. However, most of the sensor nodes are powered by batteries with limited capacities. When the batteries run out of energy, the nodes’ lifetime ends, thus creating safety hazards. It’s difficult to replace batteries for thousands of nodes, especially for the buried ones, such as pressure sensors. Fortunately, wireless charging technology, such as passive energy harvesting [1] or active power transmission [2,3,4,5], provides a promising way to solve this energy limitation problem in WSNs. The WSNs that can be recharged wirelessly are called Wireless Rechargeable Sensor Networks (WRSNs) [6].

Many ground applications have benefitted from WRSNs, such as [7,8]. In mine tunnels, the safety requirements are rigorous, and the sensor nodes are required to continue working, especially for the gas and pressure sensors. Therefore, we adopt active power transmission technologies to provide controllable and stable energy to ensure the WRSNs work perpetually. As others do, we employ several mobile chargers (MCs), traveling along tunnel tracks, to provide energy for WRSNs.

One important challenge regarding WRSNs is to design a charging schedule for sustainable network operations. Different from the applications on the ground, it is very challenging to design efficient charging scheduling algorithms for MCs in tunnels. This is because there are some constraints in tunnel environments such as: (1) MC can only travel along tunnel tracks, which means the charging path is determined beforehand; (2) all the sensor nodes are required not to “die”; (3) the number of MCs should be as low as possible since the charging device is expensive.

Although many researchers have studied the charging schedule strategies, some unsolved problems still exist. For example, the boundary conditions of the charging schedule are not derived theoretically, such as how many nodes can be supported by one MC, what the range of the charging period is, and what the minimum moving velocity of the MCs is, etc.

In this paper, considering the special tunnel scenario, we propose a periodic and Nodes-Grouped strategy to ensure all the WRSNs work perpetually. First, we divide all the WRSNs into several subnetworks and use several MCs to charge every subnetwork periodically and orderly. All the MCs only move forward, and one MC charges one subnetwork at one charging period. This mechanism ensures all the MCs move through the whole tunnel with the shortest distance, and the MC can help transport some goods for mine production at the same time. We derive a series of theorems to restrict the charging configurations for one subnetwork, to keep the entire WSN intact. It should be noted that, when charging, we use a group nodes mechanism to expand the scale of the subnetwork, for saving costs.

Specifically, our contributions in this paper include:

(1) To the best of our knowledge, this is the first attempt to give four theorems that form necessary and sufficient conditions for the complete feasibility of the charging schedule in WRSNs. Using our theorems, the charging configurations can be determined quickly and correctly.
(2) We formulate the energy replenishment and nodes grouping problem in a tunnel scenario. The problem is solved by the Complete Feasible and Nodes-Grouped Scheduling Algorithm (CFNGS).
(3) We evaluate the proposed algorithms using extensive simulations and study the impact of the multiple charging factors referred to in our theorems. The effectiveness and superiority are verified by the results.

The rest of this paper is organized as follows. Section 2 gives a brief overview of prior methods on the charging strategy of WRSNs. In Section 3, we detail the CFCS problem, boundary conditions, and the algorithm to solve CFCS. Extensive simulations and comparisons are given in Section 4, and we conclude our work in Section 5.

## 2. Related Works

The design of the charging schedule is one of the core issues in WRSNs [9]. To date, researchers have presented many excellent works to prolong the network lifetime. Although these works involve various aspects, they are mainly divided into two categories: The offline schemes and the online schemes [10].

The offline schemes are designed before charging, are usually periodic, and the MC does not change its charging decision based on a node’s information. Most of the charging schemes using MCs belong to this type. Generally, the first step of the schemes was to build the charging path according to the traveling salesman problem (TSP) [6,11], and then an optimal charging schedule would be conducted for the charging path. The number of MCs (multiple MC scheme) depends on the number of charging paths that can cover the whole network [11,12,13].

However, numerous works consider one MC in WRSNs. In [14], Fu et al., used one MC to charge all nodes in a period with the aim of minimizing charging delay. The purpose was realized by minimizing the sum of the charging time of all nodes under the constraint condition that all nodes were charged above their energy threshold. In [15], Shu et al., proposed a near-optimal velocity control strategy for MC in a WRSNs, which was periodic. They first proved the problem to be NP-hard and solved it using novel spatial and temporal discretization. In a charging period, the network lifetime was prolonged by 2.5 times by the velocity control mechanisms. The authors did not analyze the influence of nodes number and charging period, so the mechanisms cannot ensure the network works perpetually in most cases. In [16], Rao et al., considered the influence of the charging distance and angle on charging efficiency. When charging a node, they find the optimal position concerning the distance and angle for MC, which decreases the charging time by more than 50% compared with the baseline scheme. Their work is helpful for certain charging scenarios like tunnels and indoors. Similar work is detailed in [17], but the cover area of MC is further optimized by particle swarm optimization. In [18], Xu et al., proposed a novel charging model in which the nodes were partially charged, so that more sensors would be charged before their energy depletion. In [19], Wang et al., considered the recharge scheduling problem under the constraints of the vehicles’ moving energy consumption, limited recharging capacity, energy efficiency, and data latency. They employed one dedicated data gathering vehicle and multiple charging vehicles to balance energy consumption and latency. The number of vehicles was given theoretically.

The online schemes allow the MCs to change the charging behavior according to the nodes’ residual energy. In general, the nodes are charged on demand. That is to say, the nodes would not be charged unless their residual energy was below a certain threshold value. This results in the issue that if many nodes need to be charged at the same time, parts of them will stop working before being charged. Compared to the offline schemes, the online schemes have a high complexity due to continuously selecting and judging nodes to charge. However, the energy utilization efficiency of the online schemes is higher than that of the offline schemes.

In [20], He et al., proposed a charging scheme based on the Nearest-Job-Next with Preemption (NJNP) discipline and on-demand charging. The NJNP can reach a high charging efficiency by always selecting the spatially closest requesting node as the next node. In [21], Feng et al., proposed a starvation avoidance mobile energy replenishment scheme (SAMER) to solve the energy starvation problem produced in routine charging schemes, with their charging model being based on energy demand. Before charging, the maximum tolerable latency of each node would be calculated, to avoid the starvation. In [22], Lin et al., developed a Primary and Passer-by Scheduling (P^2^S) algorithm for an on-demand charging architecture for large-scale WRSNs. When choosing the charging nodes, they exploit a local searching algorithm to find primary nodes, and innovatively propose the “drawing circles” method to select passer-nodes along primary nodes. Their strategy has a high survival rate in large-scale WRSNs. In [23], Shu et al., proposed a joint energy replenishment and scheduling mechanism to maximize the network lifetime with strict sensing guarantees in WRSNs. When choosing nodes for energy portioning under the restriction of a given charging capacity of a charger, they take the operation scheduling into consideration. Then they devise an f-approximate scheduling mechanism and get a 39.2% improvement in the network lifetime over the baseline method.

To summarize, most of the charging strategies aim to prolong the network lifetime. When planning the charging strategy, most of them use a loop search method to judge the feasibility, thus leading to a low efficiency. At the same time, some scholars have proposed using massive fixed MCs to cover the interest areas [24,25]. However, this is too expensive.

Compared with the existing research, this paper provides a complete feasible charging strategy, which is constrained by four theoretically derived boundary conditions. Meanwhile, the Node-Group mechanism is put forward to enlarge the scale of the WRSNs.

## 3. The Completely Feasible and Nodes-Grouped Scheduling Algorithm

### 3.1. Problem Description

**Figure 1:**
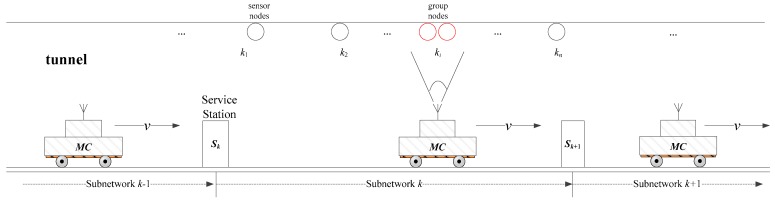
A map of a wireless rechargeable sensor networks (WRSNs).

When referring to the WRSN’s problem on the ground, researchers usually consider that the sensor nodes are distributed over a limited square area. However, in our tunnel application scenarios, the nodes present zonal distribution characteristics. Furthermore, the distribution is approximately linear because the nodes are usually fixed on the top of tunnel walls, as shown in Figure 1. In some places, the nodes are distributed sparsely, while in certain interest areas, the nodes are intensive.

Under normal circumstances, the length of the tunnel varies from dozens to thousands of meters. For a long tunnel, the number of the sensor nodes will be very large, thus it is difficult to charge the whole network using only one MC for the following two reasons: (1) it is hard to use one MC to satisfy the energy requirement of all nodes since the capacity of MC is limited; (2) when charging the front nodes, the nodes far behind may have already “died”. Therefore, in this paper, we divide the whole network into several small subnetworks. Each subnetwork has one MC, and all the MCs travel across each subnetwork one by one periodically and successively to provide power for all nodes, to perpetually prolong the lifetime of the whole network.

For each subnetwork, we consider it as consisting of several rechargeable nodes, one MC, and one service station located at the beginning. The time it takes the MC to move through the subnetwork is *T*, which is called the charging period. Here we give the definition of the charging period *T* as follows:

Definition of charging period *T*;

For a subnetwork in tunnels, if within a period time *T*, it can satisfy that;

(1) The nodes are charged during the moving of MC from one station to the next.
(2) The energy charged by the MC can ensure that all nodes will not stop working.

Then, the *T* is called a charging period *T*.

#### 3.1.1. Description of Node

We assume that all nodes are using the same hardware configuration. In a subnetwork, the set of nodes is $N=\{N_{1},N_{2},\cdots ,N_{n}\}$, in which *n* is the node’s number. Each node is equipped with a battery with maximum capacity, *E_max_*. The minimum energy level for regular operation is *E_min_*. At the beginning, the initial energy level for all nodes is *E_max_*. $p_{i}$ is denoted as the power consumption of the node *i*, and $e_{i}$ as the residual energy at the beginning of the charging round. If $e_{i}$ is below *E_min_*, the node *i* is regarded as having stopped working. In this paper, we neglect the battery degradation [26], so the *E_max_* is set as constant.

#### 3.1.2. Description of MC

A mobile charger, MC, is employed to recharge the sensor nodes. The MC mainly consists of three modules: One energy module for transferring power to nodes wirelessly, one communication module for getting node’s information (e.g., node’s location, *p_i_*, *e_i_*), one moving module for controlling the MC to travel along the tunnel. In addition, we assume that the MC foresees the power attenuation rate $\eta_{p}$ from MC to the nodes and the node rectifier efficiency $\eta_{d}$. The moving velocity of MC is *v*. When charging, the MC will stop at the nearest location of each node for affording the maximal charging power U.

#### 3.1.3. Description of Service Station

The server stations (*S*) which serve as the energy source for the MC are located at the beginning of the subnetworks. At *S*, the MC’s battery is replaced to update its energy. Compared to the node charging time, the MC’s energy updating time can be ignored. In certain scenarios, the server stations could be chosen as sink nodes for collecting sensing data. Additionally, an extra *S* is placed at the end of the last subnetwork to ensure the MC returns to the origin of the tunnel.

#### 3.1.4. Problem Statements

Our purpose is to ensure the perpetual lifetime of the whole WRSN in a tunnel. This problem can be summarized as a completely feasible charging strategy (CFCS). Based on the discussion above, the problem could be concentrated on one subnetwork. If the CFCS satisfies one subnetwork, it can be expanded to the whole network easily.

So for a subnetwork, there are two problems that must be considered:

(1) what are the boundary conditions of the CFCS?
(2) how to design the algorithm for the CFCS?

### 3.2. Problem Model

#### 3.2.1. Charging Model

In our wireless charging scenario for one subnetwork, the charging period *T* is consists of at least two segments, the total charging time of all nodes *T_c_* and the moving time of MC through the subnetwork *T_m_*.

(1) $$T_{c}=\sum_{i=1}^{n}t_{i}\;$$

*t_i_* is the respective charging time for each node.

(2) $$T_{m}=L/v\;$$

*L* is the length of the subnetwork and *v* is the moving velocity of the MC.

If the nodes do not need too much energy to be supplemented, the MC can rest at the service station when finishing the charging work. So we can get:

(3) $$T_{c}+T_{m}\leq T\;$$

During *T*, the MC must move through the subnetwork. The next round of CFCS using the next MC cannot be interrupted.

In order to make sure all the nodes won’t die from energy depletion, we must guarantee that the residual energy of each node is higher than *E_min_* at the end of *T,*

(4) $$U\cdot t_{i}-p_{i}T\geq E_{\min}\;$$

*U* is the charging power of node *i* and can be calculated by:

(5) $$U=P_{t}\eta_{p}\eta_{d}\;$$

$P_{t}$ is the transmitting power, $\eta_{P}$ is the energy transfer efficiency from MC to the sensor node in tunnel, and $\eta_{d}$ is the node rectifier efficiency.

The residual energy $e_{i}(t)$ of each node always reduces when working. The CFCS must ensure that $e_{i}(t)$ is above *E*_min_ before the MC reaching the node *i*, that means:

(6) $$e_{i}(\tau )-p_{i}(\tau )\cdot (T_{c}^{i-1}+T_{m}^{i})\geq E_{\min}\;$$

where *τ* is the time that MC reaches node *i*. $T_{c}^{i-1}$ is the total charging time of all the front *i* − 1 nodes before node *i*. $T_{m}^{i}$ is the total time generated by the moving process of MC from *S* to node *i*. $\left(T_{c}^{i-1}+T_{m}^{i}\right)$ can be calculated by:

(7) $$T_{c}^{i-1}+T_{m}^{i}=L_{i}/v+\sum_{k=1}^{i-1}t_{k}\;$$

*L_i_* is the distance between the node *i* and *S*.

We do not need to charge the battery to its full capacity, so,

(8) $$U\cdot t_{i}-e_{i}(\tau )\leq E_{\max}\;$$

and the charging power to all nodes cannot be larger than *E_MC_*,

(9) $$\sum_{i=1}^{n}U\cdot t_{i}+q\cdot L\leq E_{MC}\;$$

where *q* is the unit travel cost of MC, and in some application scenarios, *q·L* is ignored to simplify the analysis. In this paper, we ignore it too, and the Equation (9) is changed to,

(10) $$\sum_{i=1}^{n}U\cdot t_{i}\leq E_{MC}\;$$

As the transmitting power $P_{t}$ is limited, our optimal object is to find a set of charging time distribution {*t*_1_,*t*_2_,…,*t*_n_}, which satisfies the CFCS. At the same time, we maximize the total energy transferred to all nodes, that means

(11) $$\begin{array}{l} \max\sum_{i=1}^{n}U\cdot t_{i}\\ s.t.(1)-(8),(10)\\ t_{i}\geq 0 \end{array}$$

#### 3.2.2. The Boundary Conditions of CFCS

The boundary conditions satisfying CFCS are the maximum number of nodes, the range of the charging period time, and the minimum energy capacity of MC.

**Theorem 1.**  *In order to ensure the CFCS, the maximum number of nodes n should meet the relation $n<U/\bar{p}$, in which U is the charging power and $\bar{p}$ is the average power consumption among all nodes.*

**Proof.**  We set the average charging time to be $\bar{\tau }$. To ensure the CFCS, the total charging energy should be greater than or equal to the energy depletion of all *n* nodes, that is $U\bar{\tau }-n\bar{p}\bar{\tau }\geq 0$. By offsetting the $\bar{\tau }$ and performing the transposition, we can get $n\leq U/\bar{p}$. Due to the existence of MC’s moving time *T_m_*, it would lead to $U\cdot \bar{\tau }-n\bar{p}(\bar{\tau }+T_{m})\geq 0$. As long as *T_m_* exists, even if very small, the relation can be obtained that $U-n\bar{p}>0$, that is $n<U/\bar{p}$. □

**Theorem 2.**  *In order to ensure the CFCS, the range of the charging period time T should meet the relation $(1+n\bar{p}/U)T_{m}\leq T\leq (E_{\max}-E_{\min})/p_{\max}$.*

**Proof.** 

(1) We first consider the subnetwork including only one node. The maximum energy the node can be replenished by is $U\cdot T_{c}=\bar{p}T=\bar{p}(T_{c}+T_{m})$. Generally, the node cannot work when being charged. Therefore, we get $U\cdot T_{c}=\bar{p}T=\bar{p}T_{m}$, that means $T_{c}=\bar{p}T_{m}/U$. From the Equation (3), we can get $T_{m}+T_{c}=T_{m}+\bar{p}T_{m}/U\leq T$. Expand the situation to *n* nodes network, the relation $T_{m}+n\bar{p}T_{m}/U\leq T$ can be obtained. After extracting the common factor *T_m_*, we get the relation $(1+n\bar{p}/U)\cdot T_{m}\leq T$.
(2) $(E_{\max}-E_{\min})/p_{\max}$ is the ultimate working time that can be sustained when the node has the maximum power consumption. Within this time, at least once charging behavior should be implemented, otherwise the node would stop working. Thus, we can easily get that $T\leq (E_{\max}-E_{\min})/p_{\max}$.

Combining the (1) and (2) discussed above, we obtain the relation $(1+n\bar{p}/U)T_{m}\leq T\leq (E_{\max}-E_{\min})/p_{\max}$. □

**Theorem 3.**  *After the determination of T, in order to ensure the CFCS, the minimum E_MC_ should meet the relation $E_{MC}\geq P_{t}T$.*

**Proof.**  The *E_MC_* should be greater than or equal to the energy that is transferred to all *n* nodes within *T*, that is $E_{MC}\geq P_{t}T$. □

**Theorem 4.**  In order to ensure the CFCS, the MC’s velocity v should meet the relationship $v\geq \frac{nL}{2T}(\bar{p}/U+1)$.

**Proof.**  First we consider there is only one node in the subnetwork and the node’s location is at the barycenter of the subnetwork. The time consumed by MC’s moving to the node is *L*/(2*v*). For this time, the energy consumption of the node is $\bar{p}L/(2v)$. Therefore, the charging time *T_c_* is no more than $\bar{p}L/\left(2vU\right)$. According to Formula (3), we get $\bar{p}L/\left(2vU\right)+L/\left(2v\right)\leq T$. For the subnetwork with *n* nodes, it will be $n(\bar{p}L/\left(2vU\right)+L/\left(2v\right))\leq T$. After transformation, the result will be $v\geq \frac{nL}{2T}(\bar{p}/U+1)$. □

The four theorems give the boundary conditions of CFCS. Setting the parameters through these theorems, the Problem (11) would be solved, and the results could ensure that the subnetwork works perpetually. In the experiments, we use Matlab with the help of the CVX tool to solve the Problem (11). 

### 3.3. “Nodes-Grouped” Partition

**Figure 2:**
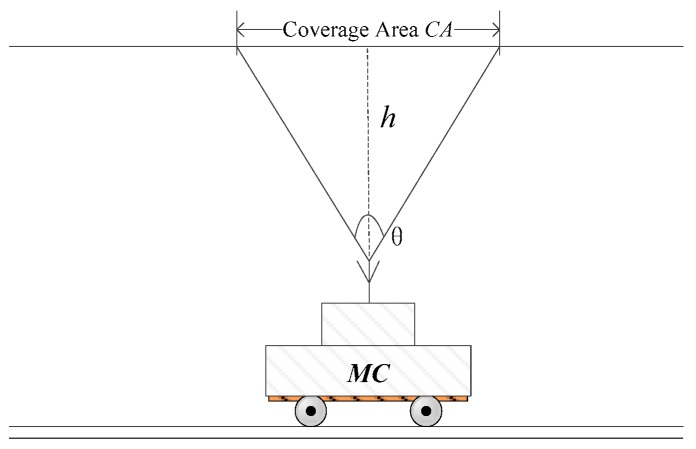
The sketch map of group node.

In the process of charging, the directional antenna with high gain is usually used in MC. Since the antenna has a beam coverage area, we can combine all nodes within it as a group node *G*. When MC moves to the center of *G*, all the nodes in *G* can be charged at one time. As shown in Figure 2, if the three nodes are all in the beam coverage area (CA), they are classified as a group node, *G_i_*. When calculating *n* using Theorem 1, *G* is treated as one node, so the real node numbers of the network can be larger than *n*.

CA is a circular region and can be obtained using the Pythagorean Theorem, as shown in Figure 2, that is $CA=\pi d^{2}\tan^{2}(\theta /2)$, where *d* is the shortest distance between the MC and the center of G. θ is the charging angle of MC.

Because *G* is treated as one node, a power consumption rate should be assigned to it. In order to make G stratify the CFCS, we assign the maximum power consumption rate of all nodes in G to G, that is $p_{G}=\max(p_{i}\in G)$.

### 3.4. Algorithm

Before dividing the whole network of the tunnel into several subnetworks, group nodes should be classified first. After that, the number of sensor nodes will be “reduced”. Then divide the subnetworks according to Theorem 1. The method of group nodes classification is simple, perform path traversal with the CA interval along the tunnel. Based on the discussion above, the whole problem can be solved by the Partition of group nodes and the Complete Feasible and Nodes-Grouped Scheduling Algorithm (CFNGS). The two algorithms are shown as below.

The time complexity of Algorithm 1 is O(m). For Algorithm 2, one charging arrangement (line 4–line 13) consists of two parts. One is solving the Semi-Definite Programming Problem (SDP, line 4), whose time complexity is $O(n^{2})$. The other is the “for” loop (line 5–line 13), whose time complexity is O(n). Therefore, the overall time complexity of Algorithm 2 is $O(n^{2})$.


**Algorithm 1. Partition of Group Nodes**
Input: the numbers of the whole nodes *m*, *L*, the nodes’ position *Ln*, *CA*
Output: new nodes’ position after group nodes classification *Ln’*, group nodes *G*1: last_id = −12: for *i* = 1:m3:   id = ceil(*Ln*(*i*)/*CA*)4:   add *Ln*(*i*) into *G*(id)5:   if id ! = last_id6:    add *Ln*(*i*) into *Ln*’7:    last_id = id8:   end if9: end10: return *Ln*’, *G*

After group nodes are portioned, the subnetworks will be divided according to Theorem 1.


**Algorithm 2. CFNGS**
Input: *n*, *E_max_*, *E_min_*, *P_t_*, $\eta_{p}$, $\eta_{d}$, $p_{i}$, *e_i_*Output: {*t*_1_,*t*_2_,…*t_n_*}1: initial medial parameters: cn = 0 % charging times,              flag = 1 % end mark for charging2: initial *T*, *E_MC_* according to Theorems 2 and 33: while (flag)4:   solve Problem (11) with CVX tool5:   for *i* = 1:*n*6:    *e*(*i*) = *E*(*i*) + *P_t_* × $\eta_{p}$ × $\eta_{d}$ × *T_w_* − $p_{i}$ × *T*; %update nodes’ residual energy7:    if *e*(*i*) ≥ *E*_min_8:      cn = cn + 1 9:      ouput {*t*_1_,*t*_2_,…*t_n_*}10:    else11:      flag = 012:    end if13:  end for14: end while

## 4. Simulations

In this section, we conduct extensive simulations and show the results to analyze the effects of different parameters on charging efficiency.

### 4.1. Simulation Settings

In our tunnel scenario, we set *m* = 100 sensor nodes which are randomly distributed along a line of 2.5 km. For each sensor node, the energy consumption rate is a value $p_{i}(t)\in (10\;\mathrm{mW},15\;\mathrm{mW})$ at node *i*. Regarding the battery capacity, we choose a regular battery with 10.8 KJ [14]. That means *E_max_* = 10.8 KJ and we let *E_min_* = 0.05 × *E_max_* = 540 J.

For the MC, it keeps moving at the speed of *v* = 5 m/s and the maximum transfer power is *P_t_* = 5 W. We assume that the shortest distance between MC and the nodes is *d* = 1 m. At this distance, we get the maximum $\eta_{p}=0.2$ from experiments in the lab. We let $\eta_{d}=0.7$ using a class-F rectifier [27]. The charging angle is θ=2π/3, from which we get *CA* ≈ 9.4 m^2^. In our simulation scenario, we set the tunnel as a line, so we should use the diameter of *CA* as the coverage area, that is *d*_*CA* ≈ 3 m.

### 4.2. Results and Analysis

#### 4.2.1. Subnetworks Partition and Nodes Grouping

**Figure 3:**
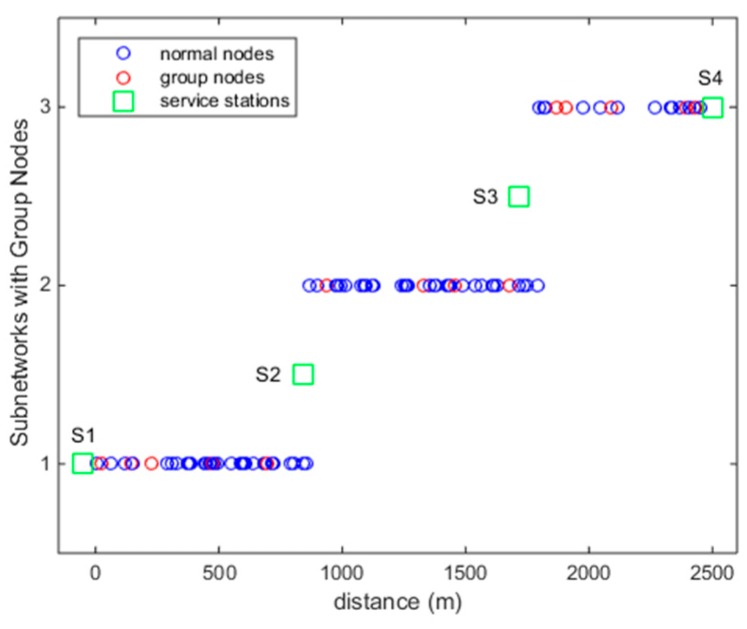
Subnetworks partition and grouping of group nodes.

From the parameters given above, the charging power is $U=P_{t}\eta_{p}\eta_{d}=0.7\;W$. According to Theorem 1, we obtain an approximate *n* < 35. Here we choose *n* = 34, so at least ⌈m/n⌉=3 interval subnetworks will be divided. Conducting Algorithm 1, the three subnetworks and group nodes are shown in Figure 3. We can see that three intervals are partitioned and group nodes are marked with the color, red. Since there are three subnetworks, at most four service stations are needed to update MC’s energy. Two are located at both ends of the whole network, and two at the junctions of intervals, shown as green squares in Figure 3. In subnetwork one, there are 38 nodes. As five groups are classified, we just consider 34 nodes when designing the scheduling strategy. In the real application, we can deploy the nodes artificially, thus forming more group nodes. Then the scale of the subnetwork will be enlarged and the number of intervals may be reduced.

#### 4.2.2. Completely Feasible Strategy

We choose the first subnetwork to simulate Algorithm 2. According to the Theorem 2, the range of the charging period is $(1+34*\bar{p}/U)\cdot L_{1}/v=317\leq T\leq (E_{\max}-E_{\min})/p_{\max}=51,300$. Here we set *T* = 10,000 s, and *E_mc_* ≥ 50,000 J according to Theorem 3. Thus far all the charging configurations are obtained as shown in Table 1.

**Figure 4:**
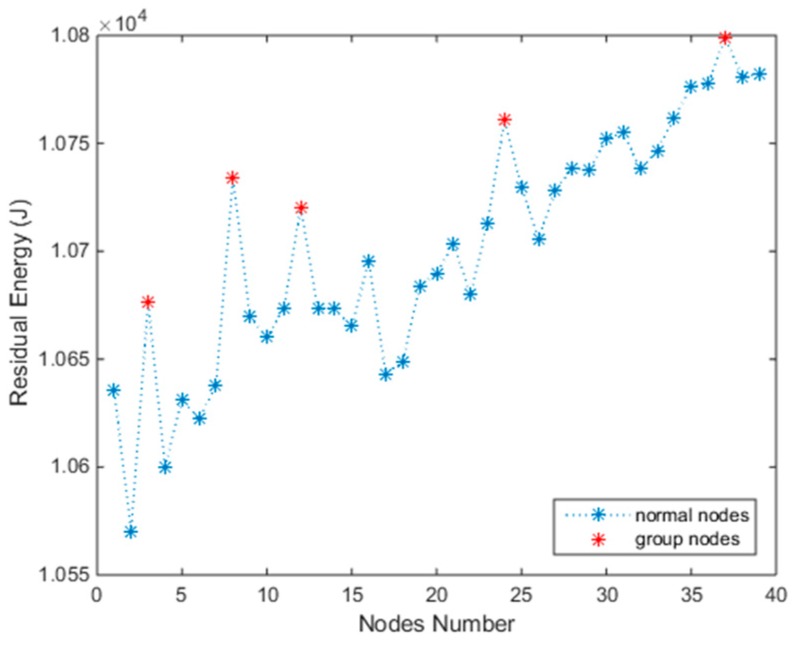
Residual energy of all nodes at balanced state.

At the initiation of charging, an MC stays at station S1 and all the Nodes’ capacity are *E_max_*. Then we begin to solve Problem (11) using Algorithm 2. An optimal set of charging times *T_c_* = {*t*_1_, *t*_2_,…, *t*_3__4_} will be output. Then the MC charges all nodes keeping to *T_c_*. When the first period *T*_1_ ends, the next MC from S1 performs the next charging task. The former MC will be used in subnetwork 2 after the energy is renewed in S2. After several *T*, the residual energies of all nodes reach a balanced condition, on which the charging time *T_c_* scarcely changes. The residual energies are plotted intuitively in Figure 4. The normal nodes’ residual energy is relatively balanced, and the latter nodes have more energy than the front nodes as they were supplemented recently. It indicates that the subnetwork 1 will work perpetually, and the results of the other two subnetworks are the same since they use the same charging schedule.

In subnetwork 1, each nodes group has two nodes. We marked the group number with a symbol “+”. For example, in Table 2, the nodes with number 2 and 2+ are in the same group, and when solving the schedule, they are treated as one node, Number 2. First, we can see that the residual energies of all nodes are more than *E_min_*, which indicates that the network works perpetually. However, at the same time, the group nodes’ residual energies are all reaching *E_max_*, which are marked with a red color in Table 2 and Figure 4. This indicates that they are overcharged. The reason for this is simple, as introduced in Section 3.3, we know that $p_{G}=\max(p_{i}\in G)$. With the same charging time, the nodes with low consumption would inevitably obtain more energy than needed, thus resulting in the overcharging phenomenon. If we set $p_{G}=\min(p_{i}\in G)$ the overcharge will disappear but group nodes with high consumption will “die” as they are not supplemented with enough energy within the same charging time. The overcharge and “dead” phenomenon will appear simultaneously when we are setting $p_{G}=\mathrm{average}(p_{i}\in G)$ because the energy radiating from MC won’t be intelligently distributed to nodes on their demands. In our next paper, we will figure out this problem with beamforming technology.

Although some nodes are overcharged, the purpose of perpetual functioning of the network is realized.

#### 4.2.3. Influence of Parameters

**Figure 5:**
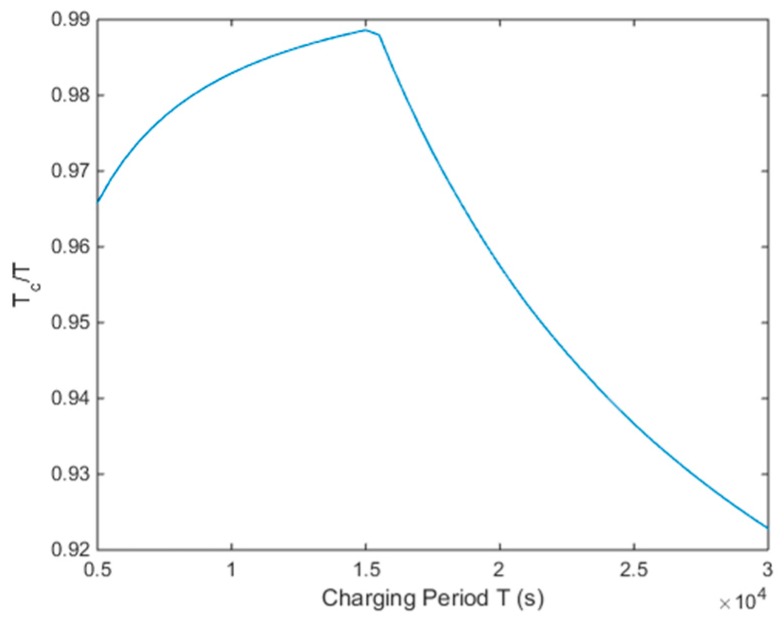
The trend of *T_c_*/*T* changes with *T.*

The charging period *T* must satisfy Theorem 2. Otherwise, the Problem (11) is unsolvable. Even when solvable, after a few charging cycles some nodes also go “dead”. The value of *T* should be based upon actual need in a real application. A smaller *T* means high frequency charging behavior, and vice versa. *T_c_*/*T* represents the rate of effective charging time, and its varying trend with *T* is shown in Figure 5. In Figure 5, we set the range of *T* from 5000 s to 30,000 s. It shows that *T_c_*/*T* initially rises and then descends with increasing *T*. The segment point is *T* = 15,000 and at that point, the whole charging system possesses the highest system efficiency. When the *T* is very large, the MC will rest longer at the service station.

**Figure 6:**
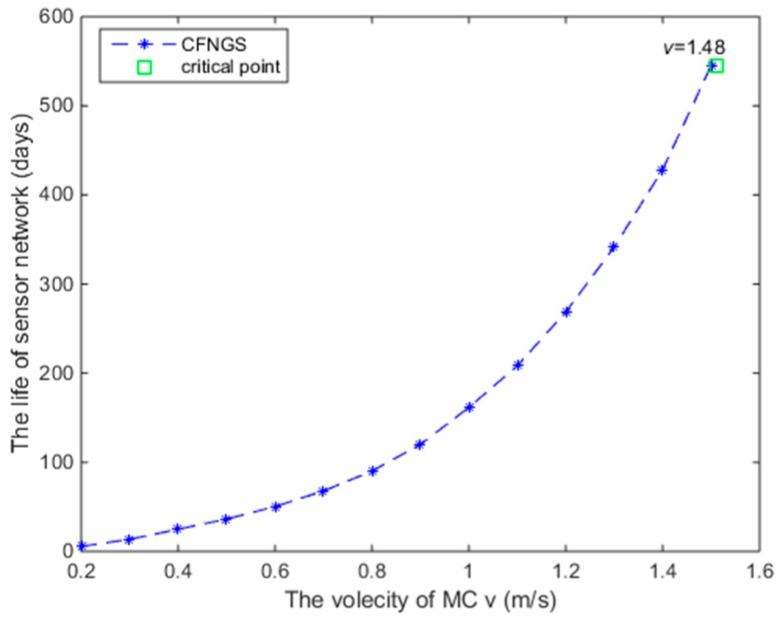
Lifetime of the subnetwork as a function of *v.*

According to our configuration and Theorem 4, the value of *v* should satisfy *v* ≥ 1.48 m/s. When *v* is set below 1.48 m/s, for example *v* = 1 m/s, the Problem (11) can still be solved. However, 189 days later, the lifetime of the subnetwork will end. That is to say, the CFCS is invalid. The lifetime of the subnetwork as a function of *v* is shown in Figure 6, from which we see that the lifetime of the subnetwork is clearly monotone, increasing with *v* before the boundary point *v* = 1.48.

**Figure 7:**
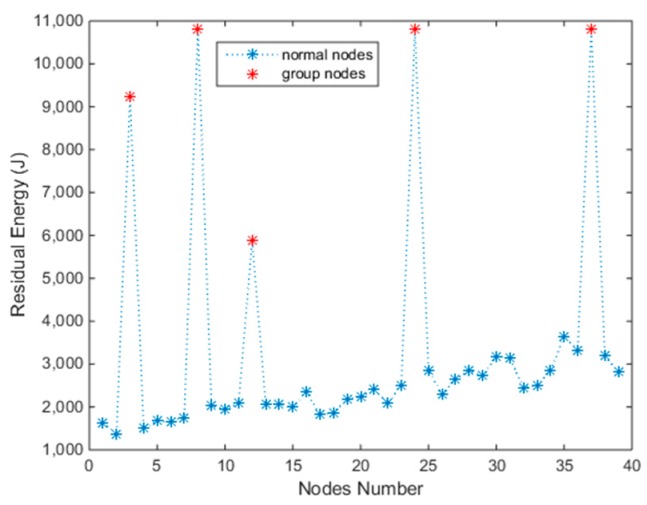
The residual energy of nodes after 391 charging cycles.

In order to satisfy CFCS, the nodes number of the subnetwork *n* should be no more than $U/\bar{p}$ according to Theorem 1. Indeed, when *n* is larger than $U/\bar{p}$, the Problem (11) may still be solved. However, after many charging cycles, some nodes will die when the residual energy is below *E_min_*. For example, we use the simulation configuration in Section 4.2, but expand *n* to 40. After 391 charging cycles, (391 charging cycles mean 391 × *T*/3600/24 = 45.25 days), the residual energies of all nodes are shown in Figure 7. At the next *T*, the first node will stop working as its residual energy is below *E_min_*. Therefore, the lifetime of this network comes to an end. From Figure 7, we can see that the residual energy of group nodes is still high, especially the nodes with lower consumption.

Without supplementing the energy, the network can only work for *E_max_*/0.025/3600/24 = 5 days. That is to say, the lifetime of the sensor network has been prolonged a further 40 days (800%), although the charging schedule does not maintain the functioning of the network perpetually. For short-term applications, the constraint of Theorem 1 could be relaxed. In addition, we can replenish the energy of all nodes to *E_max_* again before the 45th day, which can also ensure the network functions perpetually. In some cases, this mechanism can reduce the numbers of subnetworks when partitioning the whole network, thus cutting the charging cost.

The duration of lifetime is an important performance metric for different algorithms. Here we compare CFNGS with the mechanisms: Charging on demand and charging fully. Charging on demand means the node will not be charged unless it sends a charging request to the MC, at which point, its residual energy is close to *E_min_*. The charging fully mechanism means the MC charges every node to its maximum energy capacity, *E_max_*.

**Figure 8:**
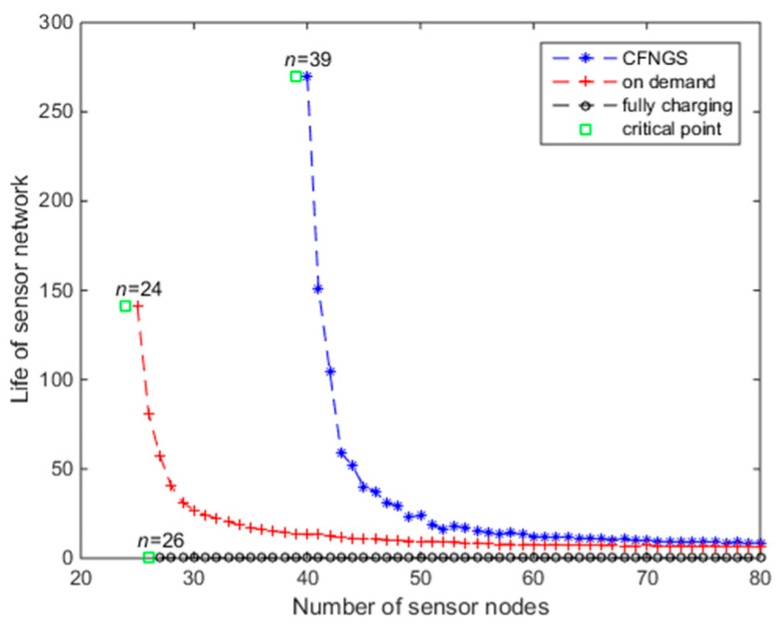
Lifetime of network changes with *n.*

The lifetime of the network, supplemented by different algorithms, as a function of *n* is shown in Figure 8. The green square markers indicate the maximum nodes numbers that different algorithms can support to ensure CFCS. For example, when *n* ≤ 39, our CFNGS algorithm could ensure that the subnetwork functions perpetually. However, the maximum nodes numbers for the charging on-demand mechanism is *n* ≤ 24, and for the charging fully mechanism is *n* ≤ 26. It clearly shows that our CFNGS algorithm can support more nodes in a subnetwork than the other two algorithms. Even with the same *n*, for example *n* = 50, CFNGS outperforms others.

The reasons for this are: Under the charging on-demand mechanism, if a many of nodes (whose residual energy is close to *E_min_*) send charging requests at the same time, some nodes will “die” before the MC goes to charges them. This mechanism is suitable for environments where the charging requirement is less urgent.

When using the charging fully mechanism, it would cost more time to charge every node to *E_max_*. The situation is the same as the charging on-demand mechanism. When lots of nodes need to be charged, the later nodes in the charging queue may “die”.

Our CFCS mechanism hardly let the nodes’ residual energy approach *E_min_*, and does not charge the nodes’ energy to *E_max_*. As a result, this mechanism can support more nodes and keep the WRSNs working longer.

For the velocity of the MC, it just needs 500 s to travel through the tunnel (*L* = 2500 m, *v* = 5 m/s). Upon comparing this with the charging period *T*, it can be ignored.

For the capacity of the MC, *E_MC_*, it must follow the constraint of Theorem 3. If the *E_MC_* is large enough to support one more subnetwork, the numbers of the service stations can be reduced accordingly.

## 5. Conclusions and Future Work

In this paper, we studied the problem of maintaining a network lifetime perpetually in a tunnel scenario, where several Mobile Chargers (MCs) travel through the tunnel track and charge the sensor nodes in a WRSN. We formulated the main problem as a charging time distribution problem subject to some charging constraints in tunnels. A complete feasible and Nodes-Grouped scheduling algorithm was put forward for this problem and the boundary conditions of CFCS were derived theoretically. We conducted extensive simulations to evaluate the performance of the proposed algorithms. The results demonstrate the correct CFCS boundary conditions and that our proposed CFCS can keep the WRSN functioning perpetually. Furthermore, our Nodes-Grouped mechanism can support more nodes in WRSN compared to some baseline methods. In the future, we will study the beamforming technology to solve the overcharge problem generated by the Nodes-Grouped mechanism.

## Figures and Tables

**Table 1:** Simulation parameters of the charging configuration.

Parameters	Values
Numbers of Nodes: *m*	100
Numbers of Nodes in Subnetwork 1: *n’*	39
Numbers of Nodes in Subnetwork 1 After Group Noding: *n*	34
The Size of Interval Network One: *L*_1_	854 m
Energy Consumption Rate of Nodes: *p_i_*	0.015–0.025 W
Average Energy Consumption Rate: $\bar{p}$	0.02 W
Coverage Area of MC: *CA*	3 m
Charging Efficiency: $U=P_{t}\eta_{p}\eta_{d}$	0.7 W
The Maximum Capacity of Battery: *E*_max_	10,800 J
The Minimum Capacity of Battery: *E*_min_	540 J
Charging Period: *T*	10,000 s

**Table 2:** Nodes information in subnetwork one in the first *T.*

Node Number	Distance (m)	*p_i_* (mW)	*T_i_* (s)	*e_i_*
1	3	16.4	234.99	10,635
2	24	23.5	336.14	10,570
**2+**	**27**	**23**	**336.14**	**10,800**
3	62	21.2	303.15	10,600
4	122	18.5	264.42	10,631
5	142	20.1	287.61	10,622
**5+**	**144**	**17**	**287.61**	**10,800**
6	154	19	271.68	10,637
7	229	15.8	225.14	10,669
**7+**	**230**	**16**	**225.14**	**10,800**
8	290	17.4	248.56	10,660
9	308	16.2	231.90	10,673
10	328	16.8	240.56	10,673
11	370	17.4	248.56	10,673
12	380	19.2	273.90	10,665
13	385	15.5	221.38	10,695
14	440	24	343.25	10,643
15	448	24.4	349.26	10,648
16	464	19.9	284.41	10,684
17	469	19.9	284.18	10,689
**17+**	**472**	**18**	**284.18**	**10,800**
18	483	18.4	262.53	10,703
19	495	24	342.86	10,680
20	553	18.7	267.03	10,713
21	588	16.1	230.17	10,729
22	592	22.8	325.75	10,705
23	604	18.9	269.96	10,728
24	608	17.4	248.81	10,738
25	642	19	271.99	10,737
26	683	16	228.06	10,752
27	687	16.3	233.14	10,754
28	697	24.4	348.86	10,738
**28+**	**698**	**15**	**348.86**	**10,800**
29	712	24.6	350.88	10,746
30	719	20.8	296.46	10,762
31	791	15.6	222.83	10,776
32	805	17.3	247.83	10,777
33	841	18.5	264.74	10,780
34	854	23.2	331.60	10,782
